# Selected Australian *Terminalia* Species Extracts Inhibit β-Lactam Drug-Resistant Bacteria Growth and Potentiate the Activity of Conventional Antibiotics: Bioactivities and Phytochemistry

**DOI:** 10.3390/microorganisms12030498

**Published:** 2024-02-29

**Authors:** Muhammad Jawad Zai, Matthew James Cheesman, Ian Edwin Cock

**Affiliations:** 1 Centre for Planetary Health and Food Security, Griffith University, Brisbane, QLD 4111, Australia; muhammadjawad.zai@griffithuni.edu.au; 2 School of Environment and Science, Griffith University, Brisbane, QLD 4111, Australia; 3 School of Pharmacy and Medical Sciences, Griffith University, Southport, QLD 4222, Australia; m.cheesman@griffith.edu.au

**Keywords:** *Terminalia ferdinandiana*, *Terminalia grandiflora*, *Terminalia microcarpa*, *Terminalia muelleri*, Kakadu plum, ESBL, MRSA, antibiotic resistance, flavonoid, tannins

## Abstract

*Terminalia ferdinandiana* Exell, *Terminalia grandiflora* Benth., *Terminalia microcarpa* Decne., and *Terminalia muelleri* Benth. (family: Combretaceae) belong to the genus *Terminalia*. Plants of this genus have been extensively used as traditional medicines to treat a variety of illnesses, including pathogen infections. However, we were unable to find any studies that have investigated the antibacterial activity of *T. microcarpa*. Similarly, whilst some preliminary studies have examined the antimicrobial properties of *T. muelleri* and *T. grandiflora*, they did not test the extracts against antibiotic-resistant pathogens. This study screens the antimicrobial activity of *T. grandiflora*, *T. microcarpa*, and *T. muelleri* and compares it to that of *T. ferdinandiana* extracts prepared from both the fruit and leaves against a range of pathogens, including multi-antibiotic-resistant strains. Solvents with varying polarities were used to extract different phytochemical constituents from the leaves of *T. grandiflora*, *T. microcarpa*, and *T. muelleri* and from the fruit and leaves of *T. ferdinandiana*. The aqueous and methanolic extracts each displayed significant antimicrobial activity when tested against the bacterial pathogens, including against the multidrug-resistant strains. When these extracts were tested in combination with selected antibiotics, some extracts potentiated the antimicrobial activity. This study identifies twelve synergistic, fifty-eight additive, and sixty non-interactive combinations, as well as thirty antagonistic effects. The extracts were evaluated for toxicity using the *Artemia franciscana* nauplii lethality assay (ALA) and were each classified as non-toxic, with the exception of the methanolic and aqueous *T. ferdinandiana* fruit extracts and the aqueous and ethyl acetate *T. ferdinandiana* leaf extracts. Metabolomic analysis using liquid chromatography–mass spectrometry (LC-MS) highlighted several flavonoids and tannins that may contribute to the antimicrobial activities reported herein. The potential antibacterial mechanism(s) of the *T. ferdinandiana* extracts are discussed in this study.

## 1. Introduction

Bacterial resistance to antibiotic therapies (AMR) is a serious threat to public health. Indeed, a 2016 AMR review authorized by the British Government claimed that if left unaddressed, AMR may result in 10 million mortalities annually by 2050 [1]. As such, the World Health Organization (WHO) has classified AMR as perhaps the most urgent threat to human health and recommended increased medical research and the adoption of a global plan to mitigate the effects of AMR [2]. Another study reported that AMR currently takes more lives than any other infectious disease, including malaria and human immunodeficiency virus (HIV) [3]. One in eight deaths worldwide is due to bacterial infections, making this the second leading cause of death after heart-related disease [4]. In 2019, AMR-associated deaths were estimated to be 4.95 million [3]. The six bacterial pathogens most frequently associated with the development of antibiotic resistance (*Escherichia coli*, *Klebsiella pneumoniae*, *Staphylococcus aureus*, *Pseudomonas aeruginosa*, *Acinetobacter baumannii*, and *Streptococcus pneumoniae*) caused approximately 3.57 million deaths in 2019 [3]. The number of deaths attributable to methicillin-resistant *Staphylococcus aureus* (MRSA) in that year was over 100,000 [3], while multidrug-resistant (MDR) tuberculosis; third-generation cephalosporin-resistant strains of *K. pneumonia* and *E. coli*; carbapenem-resistant strains of *K. pneumonia* and *A. baumannii*; and fluoroquinolone-resistant *E. coli* each caused 50,000–100,000 deaths in 2019 [3].

There has been a significant increase in research to develop novel treatments against MDR and extensively resistant pathogens. One of the suggested strategies involves the combination of potentiating molecules with ineffective (or reduced efficacy) antibiotics to restore/improve antimicrobial activity [5,6]. Some phytochemicals exhibit potent antimicrobial activities alone or when tested in combination with some clinical antibiotics, against a wide range of pathogens [7,8]. The genus *Terminalia* consists of 200–250 species, many of which have traditional uses in the treatment of a myriad of diseases and illnesses [9]. This includes (but is not limited to) treating bacterial infections, skin diseases, heart diseases, gastric ulcers, headaches, and diarrhea [10]. Phytochemical investigations into several *Terminalia* species have identified several classes of phytochemicals, including triterpenoids and their glycoside derivatives, tannins, flavonoids, and other phenolic compounds [11]. *Terminalia ferdinandiana* Exell. is an endemic Australian species that has an exceptionally high antioxidant content [12]. Indeed, the ascorbic acid levels of the fruit of this plant are substantially higher than those quantified for any plant globally [13]. Notably, previous studies have shown that *T. ferdinandiana* extracts prepared using both fruit and leaf materials inhibit MRSA growth, and it was postulated that some notable phytochemicals identified in these extracts may contribute to the therapeutic potential of *Terminalia* spp. [14]. Despite these previous studies, the therapeutic potential of *Terminalia grandiflora* Benth., *Terminalia microcarpa* Decne., and *Terminalia muelleri* Benth. remain largely unexplored.

This study assesses the antimicrobial activity of *T. grandiflora*, *T. microcarpa*, and *T. muelleri* and compares their activity with that of *T. ferdinandiana* fruit and leaf extracts. Solvents of different polarities, such as methanol, water, and ethyl acetate, were used to extract active phytochemical constituents. This study focuses on β-lactam antibiotic resistance. The bacterial species included in this study were *E. coli* and ESBL *E. coli*; *K. pneumoniae* and ESBL *K. pneumoniae*; and *S. aureus* and MRSA. Notably, whilst the bacteria studied herein are classified on the basis of their β-lactam resistance, they also exhibit resistance to several other classes of antibiotics [14]. Additionally, the antibacterial activity of *T. grandiflora*, *T. microcarpa*, *T. muelleri*, and *T. ferdinandiana* extracts was also tested in combination with conventional antibiotics to determine whether these extracts can potentiate the activity of antibiotics when used in combination, thereby repurposing those antibiotics for clinical use. Compounds were separated using high-performance liquid chromatography–mass spectrometry (HPLC-MS) for the most promising extracts. The toxicity of each extract was evaluated using *Artemia franciscana* nauplii lethality toxicity assays (ALAs). 

## 2. Materials and Methods

### 2.1. Materials

All chemicals and reagents used in this study were purchased from Ajax Fine-Chemicals Ltd., Taren Point, NSW, Australia, and were of an Analytical Research (AR) grade unless otherwise stated. Bacterial growth media was prepared using Mueller–Hinton broth and agar powders (Oxoid Ltd., Thebarton, SA, Australia) according to the manufacturer’s instructions. All other reagents were purchased from Sigma-Aldrich (Coopers Plains, QLD, Australia) unless otherwise stated. 

### 2.2. Plant Collection and Extraction 

The leaves of *T. grandiflora*, *T. microcarpa*, and *T. muelleri* were supplied by Dr Phurpa Wangchuk from the James Cook University Cairns campus. The fruit and leaves of *T. ferdinandiana* were provided by David Boehme from Wild Harvest, Northern Territory, Australia. All leaf and fruit materials were ground into a fine powder after drying in a Sunbeam food dehydrator. Voucher specimens of each plant are stored individually at Griffith University, Nathan, QLD, Australia, and are summarized in Table 1. Extraction was performed by adding 50 mL of either sterile deionized water, methanol, or ethyl acetate to the individual Falcon tubes, each containing one gram of dried and powdered plant material. The extraction of the plant material was performed via maceration for at 23 °C for 24 h. The extracts were filtered through Whatman No. 54 filter paper to remove the particulates from the extracts and dried in a vacuum oven. The mass of dried extract was measured to determine extraction yields. DMSO (100 µL) was used to partially dissolve the extract pellet, and the total volume was increased to 10 mL using sterile deionized water. The extracts were filtered using a syringe-driven filter (0.22 µm; Millipore Australia Ltd., North Ryde, NSW, Australia) and stored at 4 °C until further use.

### 2.3. Antibacterial Studies 

#### 2.3.1. Bacterial Strains Screened

This study examined the effects of the extracts against β-lactam-susceptible bacterial strains and against their antibiotic sensitive counterparts. References of ESBL β-lactam-resistant *Klebsiella pneumoniae* (ATCC 700603) and methicillin-resistant strain *Staphylococcus aureus* (MRSA) (ATCC 43300) were purchased from the American Type Culture Collection (ATCC). An ESBL-resistant *Escherichia coli* clinical isolate was supplied by the Gold Coast University Hospital, Australia. The antibiotic susceptibility of these strains towards multiple antibiotics, including β-lactam, has previously been verified in our laboratory [14,15]. As a comparison, antibiotic-susceptible strains of *E. coli* (ATCC 25922), *K. pneumoniae* (ATCC 31488), and *S. aureus* (ATCC 25923) were used in this study. 

#### 2.3.2. Growth of Bacterial Cultures

Bacterial stock cultures were initially streaked onto the Mueller–Hinton agar plates and incubated at 37 °C for 24 h to obtain pure cultures. A single bacterial colony was then transferred into freshly prepared Mueller–Hinton broth (50 mL) and incubated at 37 °C until the bacteria attained a log growth phase, with the exception of MRSA, which was incubated at 35 °C. The purity of each culture was confirmed by re-streaking the bacterial culture on Mueller–Hinton agar plates.

#### 2.3.3. Disc Diffusion Assay and Liquid Microdilution Assay

Standardized Kirby-Bauer disc diffusion and liquid-phase microdilution assays were performed to evaluate the antimicrobial susceptibility and to quantify the minimum inhibitory concentration (MIC) of each bacterial strain [15]. 

### 2.4. Examination of Combinational Effects and Identifying Optimal Ratios 

The combinational effects between the plant extracts and selected antibiotics were initially examined at 1:1 ratios. MICs were evaluated as described in Section 2.3.3. Fractional inhibitory concentration (FIC) values were calculated using the formula below:

FIC (extract) = (MIC of extract in combination with antibiotic)/MIC of extract alone.

FIC (antibiotic) = (MIC of antibiotic in combination with extract)/MIC of antibiotic alone.

∑FIC = FIC (extract) + FIC (antibiotic).

∑FIC values ≤ 0.5 were classed as synergistic, >0.5–≤1.0 were termed as additive, >1.0–≤4.0 were designated as non-interactive, and values > 4.0 were categorized as antagonistic.

Synergistic combinations were subsequently examined across a range of ratios to highlight ratio(s) that produced synergistic interactions. A modified version of the protocol described in Section 2.3.3 was used. Ratios ranged from 90% antibiotic to 10%, using 10% decreasing increments (corresponding to 10% to 90% extract). The assay was performed in two independent experiments, and FIC values were calculated. Isobolograms were plotted and used to determine the synergistic ratio(s) of extract and antibiotic. 

### 2.5. Non-Targeted LC-MS Conditions for Quantitative Analysis

Studies to identify phytochemicals in the most promising extracts were undertaken using liquid chromatography–mass spectrometry (HPLC-MS) methods previously developed by our group [15]. Putative compound identification was performed through molecular annotation against mzCloud, ChemSpider, mzVault, CyanoMetDB, and Global Natural Product Social Molecular Networking (GNPS) databases, as well as through comparison with published data. 

### 2.6. Toxicity Studies 

The toxicity of the plant extracts was evaluated using *Artemia franciscana* nauplii lethality assays (ALAs) using standard methods [15]. 

## 3. Results

### 3.1. Antimicrobial Susceptibility Studies 

**Figure 1:**
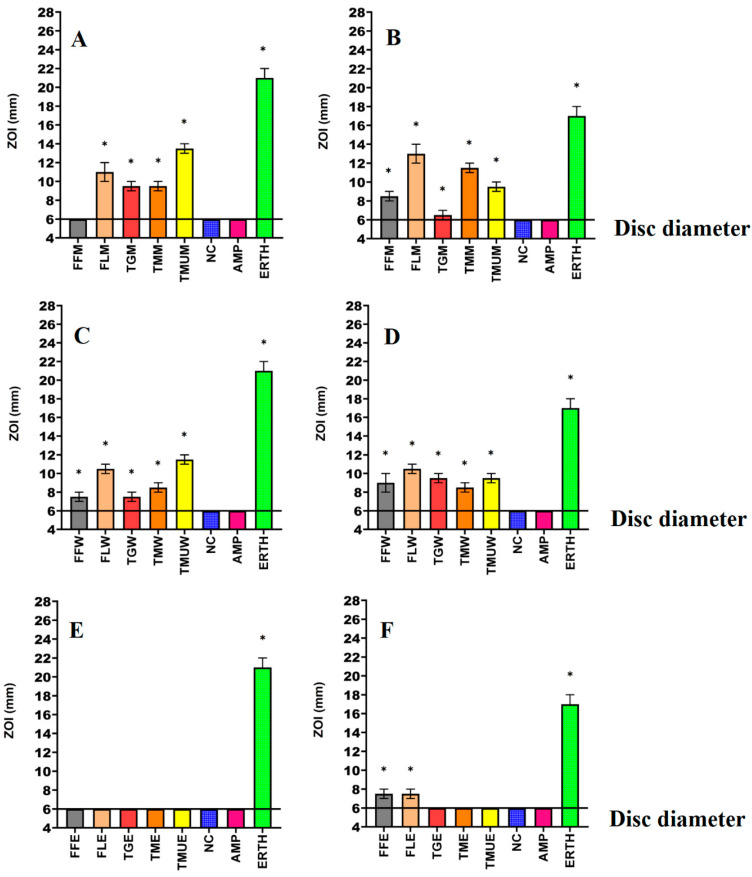
Antimicrobial effects of the (**A**) methanol extracts against *E. coli*, (**B**) methanol extracts against ESBL *E. coli*, (**C**) water extracts against *E. coli*, (**D**) water extracts against ESBL *E. coli*, (**E**) ethyl acetate extracts against *E. coli*, (**F**) ethyl acetate extracts against ESBL *E. coli*. FF = *Terminalia ferdinandiana* fruit; FL = *Terminalia ferdinandiana* leaf; TG = *Terminalia grandiflora*; TM = *Terminalia microcarpa*; TMU= *Terminalia muelleri*; M = methanol extract; W = water extract; E = ethyl acetate extract; Positive controls = ampicillin (AMP; 2 µg) and erythromycin (ERTH; 10 µg). Negative control (NC) = water. Results are expressed as mean zones of inhibition of three independent replicates ± SEM (*n* = 3). * indicates that the results are significantly different to the negative control (*p* < 0.01).

**Figure 2:**
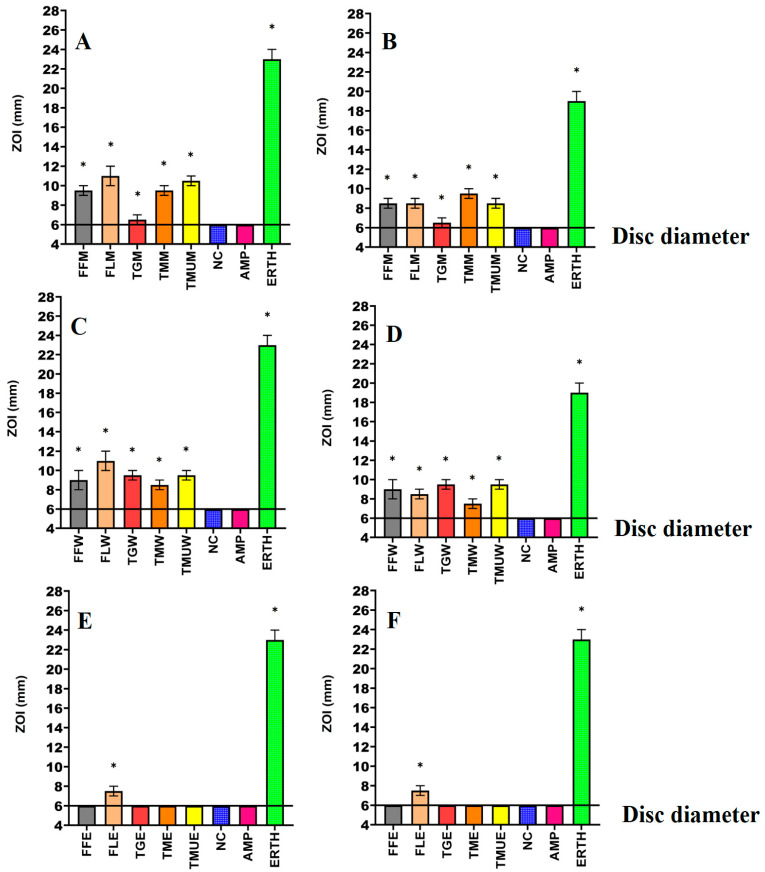
Antimicrobial effects of the (**A**) methanol extracts against *K. pneumoniae*, (**B**) methanol extracts against ESBL *K. pneumoniae*, (**C**) water extracts against *K. pneumoniae*, (**D**) water extracts against ESBL *K. pneumoniae*, (**E**) ethyl acetate extracts against *K. pneumoniae*, (**F**) ethyl acetate extracts against ESBL *K. pneumonia*. FF = *Terminalia ferdinandiana* fruit; FL = *Terminalia ferdinandiana* leaf; TG = *Terminalia grandiflora*; TM = *Terminalia microcarpa*; TMU= *Terminalia muelleri*; M = methanol extract; W = water extract; E = ethyl acetate extract. Positive controls = ampicillin (AMP; 2 µg) and erythromycin (ERTH; 10 µg). Negative control (NC) = water. Results are expressed as mean zones of inhibition of three independent replicates ± SEM (*n* = 3). * indicates that the results are significantly different to the negative control (*p* < 0.01).

**Figure 3:**
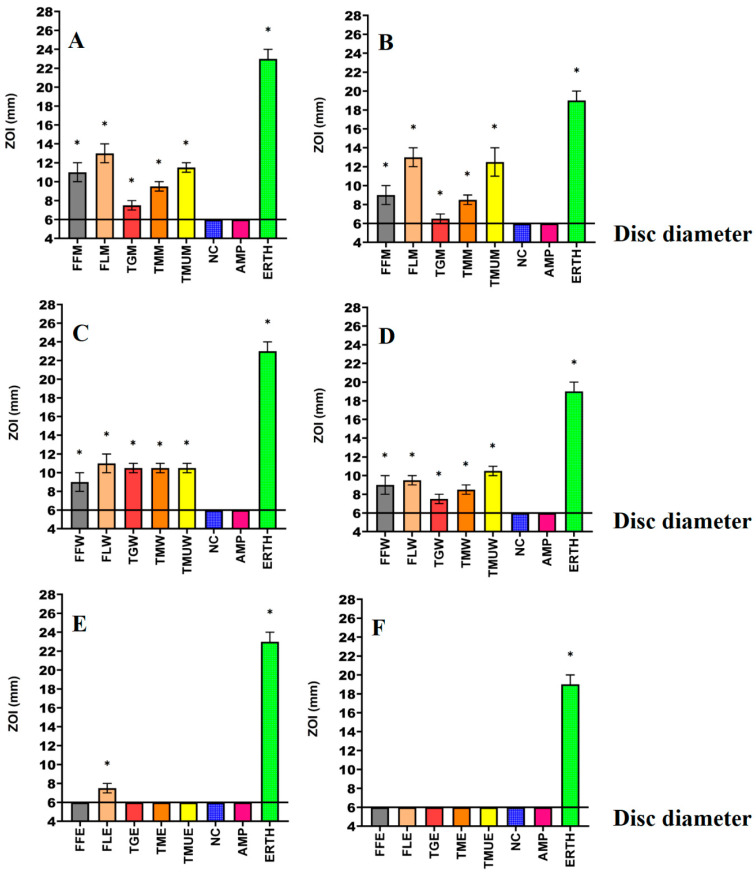
Antimicrobial effects of the (**A**) methanol extract against *S. aureus*, (**B**) methanol extracts against MRSA, (**C**) water extracts against *S. aureus*, (**D**) water extracts against MRSA, (**E**) ethyl acetate extracts against *S. aureus*, (**F**) ethyl acetate extracts against MRSA. FF = *Terminalia ferdinandiana* fruit; FL = *Terminalia ferdinandiana* leaf; TG = *Terminalia grandiflora*; TM = *Terminalia microcarpa*, TMU= *Terminalia muelleri*; M = methanol extract; W = water extract; E = ethyl acetate extract. Positive controls = ampicillin (AMP; 2 µg) and erythromycin (ERTH; 10 µg). Negative control (NC) = water. Results are expressed as mean zones of inhibition of three independent replicates ± SEM (*n* = 3). * indicates that the results are significantly different to the negative control (*p* < 0.01).

Powdered leaves of *T. grandiflora*, *T. microcarpa*, and *T. muelleri*, as well as the fruit and leaves of *T. ferdinandiana*, were extracted using solvents of different polarities and then dried and resuspended in 10 mL of 1% DMSO, resulting in the yields reported in Table 2. The antibacterial activity of each extract was first examined against *E. coli* and ESBL *E. coli* (Figure 1); *K. pneumoniae* and ESBL *K. pneumoniae* (Figure 2); and *S. aureus* and MRSA (Figure 3) in disc diffusion assays and was measured as zones of inhibition (ZOIs). The disc diffusion assays were performed to provide an estimate of bacterial infections on the solid surface. Liquid microdilution assays (Table 2) were used to quantify the growth inhibitory activity of the extracts by determining minimum inhibitory concentrations (MICs). The ethyl acetate *Terminalia ferdinandiana* leaf extract was the most potent inhibitor of ESBL *E. coli* growth (MIC = 37.5 µg/mL) and had similar activity against both antibiotic-sensitive *K. pneumoniae* and the ESBL *K. pneumoniae* (MIC = 75 µg/mL). The methanol and water extracts of *T. grandiflora*, *T. microcarpa*, and *T. muelleri* were also active, albeit with substantially lower activity. In contrast, none of the ethyl acetate extracts prepared using *T. grandiflora*, *T. microcarpa*, or *T. muelleri* leaves showed activity against any bacterial species.

### 3.2. Fractional Inhibitory Concentration

Methanol, water, and ethyl acetate extracts of *T. ferdinandiana*, *T. grandiflora*, *T. microcarpa*, and *T. muelleri* leaves, as well as *T. ferdinandiana* fruit, were combined with a range of common clinical antibiotics and tested to determine the effects of the extracts on antibiotic potency. Multiple classes of interactions were observed for combinations tested against antibiotic-sensitive and antibiotic-resistant strains of *E. coli*, *S. aureus*, and *K. pneumoniae* (Table 3). There were twelve synergistic interactions, fifty-eight combinations were determined to be additive, sixty non-interactive combinations were noted, and thirty combinations were antagonistic. As the antagonistic combinations have less efficacy compared to using either component separately, these combinations should be avoided against those bacteria. 

### 3.3. Synergistic Interaction of Extract Antibiotic at Different Ratios

**Figure 4:**
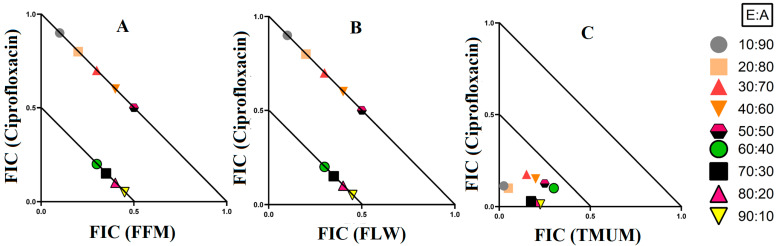
Isobologram analysis of the *E. coli* against (**A**) FFM in combination with ciprofloxacin, (**B**) FLW in combination with ciprofloxacin, (**C**) TMUM in combination with ciprofloxacin. FF = *Terminalia ferdinandiana* fruit; FL = *Terminalia ferdinandiana* leaf; TMU = *Terminalia muelleri* leaf extracts. M = methanol; W = water. FIC values are displayed as the means of two independent repeats (*n* = 2). Ratio = % extract: % antibiotic. Values below the 0.5/0.5 line represent synergy; the segment between the 0.5/0.5 and 1/1 lines represents additive interactions. Only the synergistic and additive ratios are displayed in these graphs.

**Figure 5:**
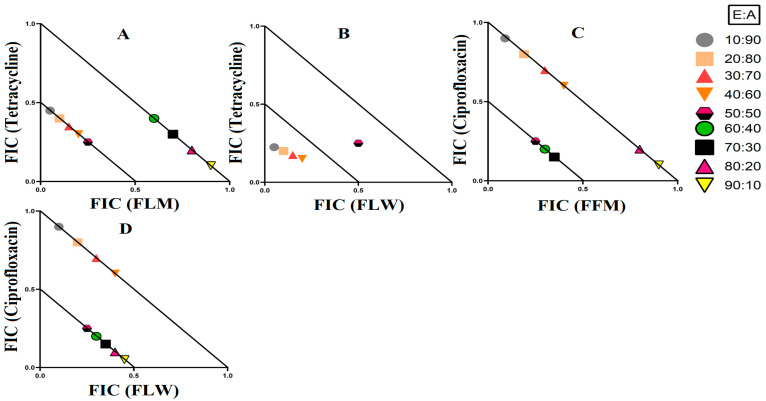
Isobologram analysis of the *S. aureus* against (**A**) FLW in combination with tetracycline, (**B**) FLW in combination with tetracycline, as well as MRSA tested against (**C**) FFM in combination with ciprofloxacin, (**D**) FLW in combination with ciprofloxacin. FF = *Terminalia ferdinandiana* fruit; FL = *Terminalia ferdinandiana* leaf; M = methanol; W = water. FIC values are displayed as the means of two independent repeats (*n* = 2). Ratio = % extract: % antibiotic. Values below the 0.5/0.5 line represent synergy; the segment between the 0.5/0.5 and 1/1 lines represents additive interactions. Only the synergistic and additive ratios are displayed in these graphs.

**Figure 6:**
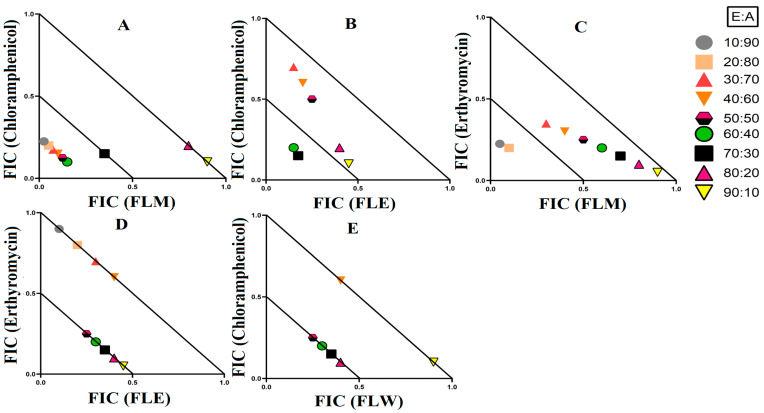
Isobologram analysis of the *K. pneumoniae* against (**A**) FLM in combination with chloramphenicol, (**B**) FLE in combination with chloramphenicol, (**C**) FLM in combination with erythromycin, (**D**) FLE in combination with erythromycin, as well as isobologram of ESBL *K. pneumonia* against (**E**) FLW in combination with ciprofloxacin. FL = *Terminalia ferdinandiana* leaf; M = methanol; W = water; E = ethyl acetate. FIC values are displayed as the means of two independent repeats (*n* = 2). Ratio = % extract: % antibiotic. Values below the 0.5/0.5 line represent synergy; the segment between the 0.5/0.5 and 1/1 lines represents additive interactions. Only the synergistic and additive ratios are displayed in these graphs.

Three synergistic interactions were noted against *E. coli* (Figure 4). Two further synergistic interactions were noted against *S. aureus*, as well as two against MRSA (Figure 5). Additionally, four synergistic interactions were noted against *K. pneumoniae* and one against ESBL *K. pneumoniae* (Figure 6). Therefore, these combinations were also tested at various ratios and graphed as isobolograms to identify the ratios that produced synergistic interactions.

#### 3.3.1. Extract and Antibiotic Synergistic Interactions against *E. coli*

There were three synergistic interactions noted against *E. coli*, all of which were in combinations containing ciprofloxacin as the antibiotic component. When *T. ferdinandiana* fruit methanol extracts were combined with ciprofloxacin, it exhibited synergistic inhibition in ratios containing 60–90% extract, while ratios containing 10–50% extracts produced additive effects (Figure 4A). The combination of *T. ferdinandiana* leaf water extract with ciprofloxacin produced synergistic interaction in ratios containing 60–90% extract and additive effects in ratios that were made up of 10–50% extracts (Figure 4B). Interestingly, the combination containing the *T. muelleri* methanol extract produced a synergistic effect at all ratios (10–90% extract; Figure 4C). Importantly, ratios with indifferent effects have no benefits compared to the use of individual components. Therefore, ratios producing indifferent effects are not shown in the isobolograms.

#### 3.3.2. Extract and Antibiotic Synergistic Interactions against *S. aureus* and MRSA 

Four extract and antibiotic combinations produced synergistic effects against the bacterial pairing of *S. aureus* and MRSA. Combinations containing either *T. ferdinandiana* leaf methanol extract or *T. ferdinandiana* water extract in combination with tetracycline produced synergistic effects against *S. aureus*. *Terminalia ferdinandiana* leaf methanol extract in combination with tetracycline produced synergy in ratios containing 10–50% extract (Figure 5A), whilst the combination of *T. ferdinandiana* leaf water extract and tetracycline yielded synergistic interactions in ratios containing 10–40% extract (Figure 5B). Two synergistic interactions were recorded against MRSA, both of which contained ciprofloxacin. Combining *T. ferdinandiana fruit* methanol extract with ciprofloxacin produced synergistic inhibition at ratios containing 50–70% extract (Figure 5C), while *T. ferdinandiana* leaf water extract in combination with ciprofloxacin induced synergy at ratios comprising 50–90% extract (Figure 5D).

#### 3.3.3. Extract and Antibiotic Interactions against *K. pneumoniae* and ESBL *K. pneumoniae*

Four synergistic interactions were recorded against *K. pneumoniae* and one against ESBL *K. pneumoniae*. The combination of *T. ferdinandiana* leaf methanol extracts with chloramphenicol produced synergy against *K. pneumoniae* at ratios containing 10–60% extract (Figure 6A). However, only two ratios (containing 60% and 70% extract) were synergistic when *T. ferdinandiana* leaf ethyl acetate was combined with chloramphenicol and tested against *K. pneumoniae* (Figure 6B). Similarly, two ratios (10% and 20% extract) were synergistically active against *K. pneumoniae* when *T. ferdinandiana* leaf methanol extracts were combined with erythromycin (Figure 6C). *Terminalia ferdinandiana* leaf ethyl acetate extract produced synergy in combination with erythromycin against *K. pneumoniae* at ratios comprising 50–90% extract (Figure 6D). Synergistic interactions against ESBL *K. pneumoniae* were recorded at ratios containing 50–80% *T. ferdinandiana* leaf water extract combined with chloramphenicol (Figure 6E). 

### 3.4. Identification of Compounds in the FLM and FLW Extracts

**Figure 7:**
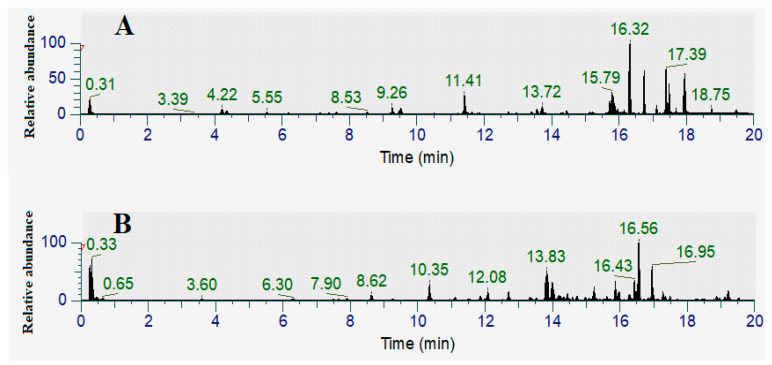
Total compound LC-MS chromatograms in positive ionization mode of (**A**) FLM (*Terminalia ferdinandiana* leaf methanol extract), (**B**) FLW (*Terminalia ferdinandiana* leaf water extract).

The aqueous and methanolic *T. ferdinandiana* leaf extracts showed the greatest antibacterial activity in the disc diffusion assays, as well as in the liquid dilution assays. Therefore, these extracts were subjected to phytochemical separation and identification studies. Optimized HPLC-MS parameters that were previously established by our group for [15] were employed to develop a metabolomic fingerprint of the aqueous and methanolic *T. ferdinandiana* leaf extracts, focusing on the flavonoids and tannins. Total compound chromatograms (TCCs) recorded in the positive ionization mode are shown in Figure 7A and Figure 7B for *T. ferdinandiana* leaf methanol and water extracts, respectively. 

In this study, we focused on the identification of flavonoids and tannins. Flavonoids are well known as an antimicrobial agent and with an increasing incidence of highly antibiotic-resistant infections, flavonoids have potential to be substituted for antibiotics [16]. Similarly, studies have also reported the use of extracts rich in tannins for treating ailments such as bacterial infections [17]. A range of flavonoids and tannins were identified in this study (Table 4). Flavonoids identified in the *T. ferdinandiana* leaf methanol extracts include vitexin, robinetin, quercitin-3β-D-glucoside, quercetin 3-O-rhamnoside-7-O-glucoside, orientin, hispidulin 7-glucuronide, hibiscetin 3-glucoside, 6-hydroxyluteolin 6-glucuronide, 4-(3,4-dihydroxyphenyl)-7-hydroxy-5-{[(2S,3R,4S,5S,6R)-3,4,5-trihydroxy-6-(hydroxymethyl)oxan-2-yl]oxy}-2H-chromen-2-one, 3,7-dihydroxy-4,5-dimethoxy-8-prenylflavan 7-O-beta-D-glucopyranoside, 4-(3,4-dihydroxyphenyl)-7-hydroxy-5-{[2S,3R,4S,5S,6R)-3,4,5-trihydroxy-6-(hydroxymethyl) oxan-2-yl]-2H-chromen-2-one, 2-(3,4-Dihydroxyphenyl)-3,5,7-trihydroxy-8-{[(2R,3R,4S,5S,6R)-3,4,5,6-tetrahydroxytetrahydro-2H-pyran-2-yl]oxy}-4H-chromen-4-one, 1,5-Anhydro-1-[5,7-dihydroxy-3-(4-hydroxyphenyl)-4-oxo-4H-oxo-4H-chromen-8-yl] hexitol, and (1ξ)-1,5-Anhydro-1-[2-(3,4-dihydroxyphenyl)-5,7-dihydroxy-4-oxo-4H-chromen-8-yl]-D-galactitol. Orientin and 1,5-Anhydro-1-[5,7-dihydroxy-3-(4-hydroxyphenyl)-4-oxo-4H-chromen-8-yl] hexitol were also identified in the *T. ferdinandiana* leaf water extract, in addition to vitexin 2″-p-hydroxybenzoate, 5,7,2′,5′-tetrahydroxy-6-methoxyflavanone, and (2S,3R,4R,5S,6S)-2-{[2-(3,4-dihydroxyphenyl)-5,7-dihydroxy-4-oxo-4H-chromen-3-yl]oxy}-3,5-dihydroxy-6-methyloxan-4-yl 3,4,5-trihydroxy-benzoate. In contrast, the tannin ellagic acid was identified in both the *T. ferdinandiana* leaf methanol and water extracts, whilst pyrogallol was only identified in the water extract. 

### 3.5. Quantification of Toxicity 

The toxicity of the extracts was determined using ALA toxicity assays. Extract dilutions that produce ≤50% mortality were deemed to be non-toxic at those concentrations. With the exception of the *T. ferdinandiana* fruit methanol, *T. ferdinandiana* fruit water, *T. ferdinandiana* leaf ethyl acetate, and *T. ferdinandiana* leaf water extracts, all other extracts were non-toxic at 1000 µg/mL. The toxic extracts were further diluted to allow for the determination of the LC_50_. The LC_50_ for *T. ferdinandiana* fruit methanol, *T. ferdinandiana* fruit water, and *T. ferdinandiana* leaf water was calculated to be 250 µg/mL, whilst the LC_50_ of *T. ferdinandiana* leaf ethyl acetate was 500 µg/mL. These extracts were therefore classified as toxic. The potassium dichromate positive control (2 mg/mL) induced 100% mortality following 24 h of exposure, whilst the seawater negative control induced 0% mortality. 

## 4. Discussion

Solvent extraction is commonly used for the isolation of phytoconstituents present in plant materials [18]. The yield of the extracts and the resulting bioactive compounds present in the plant extracts are strongly dependent on the nature of the extraction solvent, due to the presence of compounds of varied chemical characteristics that may or may not be soluble in a particular solvent. The methanolic and aqueous extracts tested in this study inhibited all of the bacterial strains tested including the antibiotic-resistant strains in the disc diffusion susceptibility assay, (Figure 1, Figure 2 and Figure 3) as well as in the liquid dilution assay (Table 2). In contrast, the ethyl acetate extracts prepared from all plant materials completely lacked inhibitory activity in the liquid dilution assay, except the FLE extract, which was highly effective against ESBL *E. coli* (37.5 µg/mL), *K. pneumoniae* (75 µg/mL), and ESBL *K. pneumoniae* (75 µg/mL). These differences may be due to the phytochemical components extracted from the leaves and fruit of plants using different solvents, with varying polarities. Higher-polarity solvents generally extract a wider variety of phytochemicals and are in greater abundance compared to solvents that are lower in polarity [19]. Additionally, phytochemicals that are larger in molecular size and/or lower in polarity may not easily diffuse through agar gels, potentially affecting the observed antibacterial efficacy of the extracts in solid-phase assays such as the disc diffusion assay [19]. The compositional differences between the extracts may explain the variation in antimicrobial activity between the solid and liquid phase assays and between the different solvent extracts. Additionally, volatile phytochemicals evaporate from the surface of agar gels, thereby decreasing their concentration in the assay and therefore decreasing the observed effectiveness [20]. Polar compounds are generally more soluble in aqueous solutions and, as a result, diffuse more rapidly through agar gels. In contrast, less water-soluble compounds may diffuse less rapidly and concentrate around the disc, which may result in underestimating the MIC values of the extracts using disc diffusion methods [21]. Broth microdilution assays are less susceptible to the impact of the compound size and polarity and are generally deemed to be better than the disc diffusion assay for quantifying antibacterial activity. 

We were unable to find previous studies that examined the antimicrobial activity of *T. grandiflora* and *T. microcarpa* against MRSA and ESBL antibiotic-resistant pathogens. Interestingly, the *T. grandiflora* water extract (TGW) had moderate activity against *E. coli* and ESBL *E. coli* (1600 µg/mL), whilst the *T. muelleri* methanol extract also had moderate growth inhibitory activity against *E. coli* (1300 µg/mL) and ESBL *E. coli* (2600 µg/mL) (Table 2). Notably, a previous study reported the antibacterial activity for an ethyl acetate extract of *T. muelleri* against *E. coli* and *S. aureus* using disc diffusion assays [22]. However, that study used a fractionated ethyl acetate extract, which was tested at a single high concentration (10 mg/mL). That study did not determine MIC values, and therefore it is not possible to compare the activity with other studies. Notably, the *Terminalia muelleri* ethyl acetate extract tested in our study lacked antibacterial activity in the disc diffusion assay. However, our study tested crude extract (3.6 mg/mL), without the fractionation and concentration steps reported in the earlier study. Therefore, the ethyl acetate extract tested in our study was of a substantially lower concentration than in the previous study. In contrast, the *Terminalia muelleri* aqueous and methanolic extracts tested herein inhibited the growth of both antibiotic-sensitive and antibiotic-resistant strains in the liquid dilution assays, with MICs as low as 1375 µg/mL against *S. aureus* (Table 2). 

Previous studies have reported antimicrobial activity for *T. ferdinandiana* fruit methanolic extracts, as well as the aqueous, methanolic, and ethyl acetate leaf extracts of this species [14]. *Terminalia ferdinandiana* fruit and leaf extracts inhibited bacterial growth in our study (Table 2). The ethyl acetate leaf extract was particularly potent against ESBL *E. coli* (37.5 µg/mL), as well as the antibiotic-sensitive and antibiotic-resistant strains of *K. pneumoniae* (75 µg/mL). The plant extracts in our study may function via a mechanism(s) that is/are distinct from those of the β-lactam antibiotics to which these strains have developed resistance. Alternatively, the phytochemical constituents present in these extracts may block bacterial antibiotic-resistance mechanisms, allowing the inhibitory components to function with increased potency. This is encouraging, as the resistant bacterial strains investigated in our study have substantially reduced susceptibilities to antibiotics from diverse classes, including aminoglycosides, β-lactams, fluoroquinolones, macrolides, sulfonamides, and tetracycline. Additionally, these extracts were effective against both Gram-positive and Gram-negative pathogens, highlighting their potential for broad-spectrum antibiotic therapy. Future studies to screen these extracts against a more comprehensive range of bacteria, including further MDR strains, are planned to comprehensively assess their potential as antibiotic therapy components. 

Synergistic combinations containing plant extracts or pure compounds are an emerging area of interest for medical research to combat bacteria that are resistant to conventional antibiotics [23]. In this study, we observed twelve synergistic, fifty-eight additive, sixty non-interactive, and thirty antagonistic interactions. Synergistic combinations significantly increase the antimicrobial effectiveness of the antibiotics compared to additive interactions and therefore have substantial potential for the development of novel and effective antibiotic therapies. Synergistic interactions were observed when *T. ferdinandiana* fruit methanol, *T. ferdinandiana* leaf water, and *T. muelleri* methanol extracts were combined with ciprofloxacin and tested against *E. coli* (Figure 4). Synergy was also observed when *T. ferdinandiana* leaf methanol and *T. ferdinandiana* leaf water extracts were combined with tetracycline and tested against *S. aureus*, whilst combining *T. ferdinandiana* fruit methanol and *T. ferdinandiana* leaf water with ciprofloxacin produced synergy against MRSA (Figure 5). Combining *T. ferdinandiana* leaf methanol and *T. ferdinandiana* leaf ethyl acetate with chloramphenicol or combining *T. ferdinandiana* leaf methanol and *T. ferdinandiana* leaf ethyl acetate with erythromycin results in synergistic inhibition of *K. pneumoniae*. Additionally, the combination of *T. ferdinandiana* leaf water and chloramphenicol was synergistic against ESBL *K. pneumoniae* (Figure 6). The plant extracts tested in this study may contain components that block the resistance mechanism of ciprofloxacin, tetracycline, chloramphenicol, and erythromycin, although this needs to be verified in future studies. In contrast, non-interactive combinations neither increase nor decrease the antimicrobial effects compared to the extract or antibiotic when tested alone, indicating that they are safe for simultaneous use, despite providing no additional benefits over using individual components alone. Antagonistic interactions reduce the antimicrobial activity of combinations and hence should be avoided. Notably, all the extracts except *T. ferdinandiana* fruit methanol, *T. ferdinandiana* fruit water, *T. ferdinandiana* leaf ethyl acetate, and *T. ferdinandiana* leaf water were determined to be non-toxic in the ALA toxicity assay.

**Figure 8:**
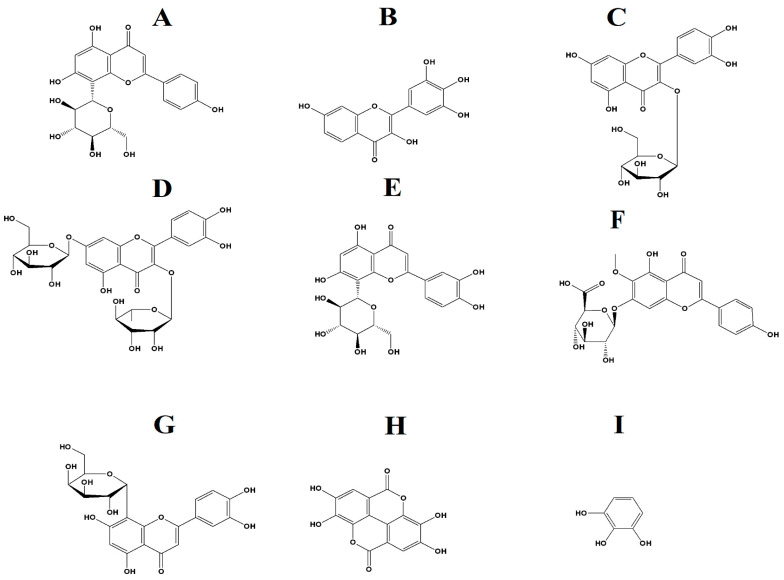
(**A**) vitexin, (**B**) robinetin, (**C**) quercitin-3β-D-glucoside, (**D**) quercetin 3-O-rhamnoside-7-O-glucoside, (**E**) orientin, (**F**) hispidulin 7-glucuronide, (**G**) (1ξ)-1,5-Anhydro-1-[2-(3,4-dihydroxyphenyl)-5,7-dihydroxy-4-oxo-4H-chromen-8-yl]-D-galactitol, (**H**) ellagic acid, (**I**) pyrogallol.

Phytochemical evaluations of several *Terminalia* spp. in previous studies have highlighted several interesting compounds, including tannins such as chebulic acid, ellagic acid, and gallic acid, as well as multiple flavonoids [24]. Several tannins and flavonoids were also identified in our study in the aqueous and methanolic *T. ferdinandiana* leaf extracts (Table 4). In particular, the flavonoids vitexin (Figure 8A), robinetin (Figure 8B), quercitin-3β-D-glucoside (Figure 8C), quercetin 3-O-rhamnoside-7-O-glucoside (Figure 8D), orientin (Figure 8E), hispidulin 7-glucuronide (Figure 8F), and (1ξ)-1,5-anhydro-1-[2-(3,4-dihydroxyphenyl)-5,7-dihydroxy-4-oxo-4H-chromen-8-yl]-D-galactitol (Figure 8G), as well as the tannins ellagic acid (7H) and pyrogallol (7I), were identified herein. Interestingly, flavonoids produce antimicrobial activities via several mechanisms, including alterations in cytoplasmic membrane function, suppressing nucleic acid synthesis, and via modulation of energy metabolism [16,25,26,27]. Flavonoids also reduce biofilm formation and adhesion, membrane permeability, and the number of porins in the cell membrane, as well as pathogenicity, which all are important factors for bacterial growth and survival [16,26,27]. Interestingly, flavonoids can also reverse antibiotic resistance in some bacteria and may also improve the effectiveness of other antibiotic components [26,27]. Hence, flavonoid-based medications may have potential for the treatment of antibiotic-resistant infections.

Two flavonoids, kaempferol 3-*O*-α-l-(2″-*Z*-*p*-coumaroyl-4‴-*E*-*p*-coumaroyl)-rhamnoside and kaempferol 3-*O*-α-l-(2‴,4‴-di-*E*-*p*-coumaroyl)-rhamnoside, which was isolated from *Laurus nobilis* L. leaves, have been shown to possess anti-MRSA activity [28]. Both compounds out-performed conventional antibiotics, including ciprofloxacin, erythromycin, norfloxacin, oxacillin, and tetracycline, against MRSA, with MIC values of 0.5–2.0 µg/mL [28]. Flavonoids isolated from a *Paulownia tomentosa* Steud. fruit ethanolic extract also possess antimicrobial activity and inhibit the growth of *Bacillus cereus*, *Bacillus subtilis*, *Enterococcus faecalis*, *Listeria monocytogenes*, *S. aureus*, and *Staphylococcus epidermidis* [29]. Flavonoids may exert antimicrobial activity by inhibiting the activity of the enzyme DNA gyrase, which is required to relieve torsional strain in the double-stranded DNA helix during bacterial replication [30]. The flavonoids quercetin and hispidulin inhibit the activity of *Mycobacterium tuberculosis* (MIC = 50 µg/mL and MIC = 100 µg/mL, respectively) [31]. Notably, the phytochemical analysis reported in our study identified quercetin derivatives (quercetin-3β-D-glucoside; quercetin 3-O-rhamnoside-7-O-glucoside), as well as a hispidulin glycoside (hispidulin 7-glucuronide) (Table 4). These compounds may contribute to the antibacterial activity of these extracts, although this needs to be verified. 

Tannins also possess an antimicrobial activity as they can pass through the cell wall of the bacteria and interfere with the cell metabolism, thereby causing their destruction [17]. Many chronic and persistent bacterial infections are linked to the formation of biofilms, with more than 60% of all microbial infections involving the formation of biofilms [32]. Biofilm forming bacteria are often difficult to treat as they can tolerate immune defenses, biocides, antibiotics, and hydrodynamic shear forces [33]. Ellagic acid, also identified in this study (Table 4), inhibits the biofilm formation of *E. coli* by 22–26% [33]. Furthermore, the combination of ellagic acid and thioridazine (an efflux pump inhibitor) can reduce the formation of biofilm by *E. coli* by between 73% and 89% [33]. Pyrogallol, also identified in our study (Table 4), has also previously been reported to have antibacterial activity, with an MIC in the range of 32–64 µg/mL against *Vibrio parahaemolyticus* [34]. Additionally, 5 mM and 10 mM concentrations of pyrogallol were reported to inhibit the growth of *Pseudomonas pyocyanea*, *Pseudomonas putida*, and *Corynebacterium xerosis* in disc diffusion assays [35]. However, the antimicrobial activity of pyrogallol against MRSA and ESBL-producing bacterial strains has not yet been verified. 

The flavonoids and tannins identified in this study may possess inherent antimicrobial activities by themselves, and/or they may potentiate the activity of other phytochemicals present in the extract. However, further studies are required to examine their effects and their antimicrobial mechanisms. Studies should also explore the potential of these compounds for new antibiotic therapies or as potentiators of current antibiotics. The extracts tested herein were effective against both the antibiotic-sensitive strains and their antibiotic-resistant counterparts, a desirable trait for new and effective antibiotics. Notably, some compounds were unable to be identified using LC-MS metabolomic profiling, and these compounds may contribute to the antibacterial activity noted in our study (either directly or as potentiators). Furthermore, volatile and low-polarity compounds may also have evaporated during the assay and/or extraction process. Hence, further phytochemical studies should utilize different methods such as GC-MS for a complete evaluation of the extract composition. *Artemia* nauplii toxicity assays were used to evaluate the toxicity of the extracts, with the majority of the extracts determined to be non-toxic. However, further toxicity evaluations of the extracts against an extensive panel of human cell lines are needed to confirm their low toxicity and hence their safety for medicinal use. 

## 5. Conclusions

The urgent need to address antimicrobial resistance has led to a significant increase in the number of studies testing natural products and extracts as potential sources to develop new antibiotics or to increase the efficacy of current clinical antibiotics. The plant extracts examined in our study inhibited ESBL and MRSA bacterial growth as effectively as they inhibited the antibiotic-susceptible strains. This indicates that the phytochemicals present in these extracts may have novel and/or uncharacterized antibacterial mechanisms. Future studies should investigate the antimicrobial properties of these compounds and their ability to potentiate the activity of conventional antibiotics. 

## Figures and Tables

**Table 1:** Source and voucher numbers for plant specimens used in this study.

Species	Common Name	Plant Part	Origin	Supplier	Voucher Number
*Terminalia ferdinandiana* Exell.	Kakadu plum, gubinge, billygoat plum	Fruit	Kakadu Nation Park, Northern Territory, Australia (collected under licence)	David Boehme, Northern Territory Wild Harvest	GU_NT_TFF21
		Leaves			GU_NT_TFL21
*Terminalia gradndflora* (Benth.) Kuntze	Yalu, plumwood, nutwood	Leaves	James Cook University, Cairns campus, Australia	Dr. Phurpa Wangchuk, James Cook University, Australia	GU_NQ_TGrL21
*Terminalia microcarpa* Decne.	Damson plum, sovereign wood	Leaves	Kimberley Wild Gubinge, Western Australia	Jacinta Monck, Kimberley, Australia	GU_WA_TmicL21
*Terminalia muelleri* Benth.	Australian almond	Leaves	James Cook University, Cairns campus, Australia	Dr. Phurpa Wangchuk, James Cook University, Australia	GU_NQ_TMueL21

**Table 2:** Yield (mg/mL) and MIC values (µg/mL) of plant extracts and conventional antibiotics against the bacteria tested in this study.

Extract and Antibiotic	MIC (µg/mL)						
	*E. coli*	ESBL *E. coli*	*S. aureus*	MRSA	*K. pneumoniae*	ESBL *K. pneumoniae*	Yield (mg/mL)
FFM	1344	1344	-	1344	-	-	21.5
FLM	**498**	**996**	**996**	**996**	**996**	**996**	31.9
TGM	2550	2550	-	2550	2550	2550	10.2
TMM	1300	2600	1300	2600	2600	2600	20.8
TMUM	3400	3400	1700	6800	3400	3400	27.2
FFW	**606**	1212	**606**	1212	1212	1212	38.8
FLW	**663**	**331**	**165**	**663**	**331**	**331**	10.6
TGW	1600	1600	3200	1600	3200	3200	12.8
TMW	2063	4125	2063	2063	2063	2063	16.5
TMUM	2750	5500	1375	2750	2750	2750	22
FFE	-	1225	-	-	-	-	4.9
FLE	-	**37.5**	-	-	75	75	0.3
TGE	-	-	-	-	-	-	7
TME	-	-	-	-	-	-	6.6
TMUE	-	-	-	-	-	-	3.6
Tetracycline	-	-	1.25	-	-	-	
Chloramphenicol	-	-	0.31	-	1.25	1.25	
Ciprofloxacin	2.5	-	0.62	2.5	2.5	1.25	
Gentamicin	0.039	0.039	0.03	0.03	0.03	0.03	
Erythromycin	-	-	1.25	-	2.5	-	
Negative control	-	-	-	-	-	-	

Yield and MIC values for FF = *Terminalia ferdinandiana* fruit; FL = *Terminalia ferdinandiana* leaves; TG = *Terminalia grandiflora*; TM = *Terminalia microcarpa*; TMU = *Terminalia muelleri;* M = methanol extract; W = water extract; and E = ethyl acetate extract. Noteworthy MIC values (<1000 μg/mL) are highlighted in bold. - indicates no inhibition was observed at any concentration tested. MIC values of triplicate determinations (*n* = 3) are shown and are expressed in units of µg/mL.

**Table 3:** ∑ FIC values for interactions between plant extracts and antibiotics.

Bacteria	Extract	Tetracycline	Chloramphenicol	Ciprofloxacin	Gentamicin	Erythromycin
*E. coli*	FFM	-	-	**0.50**	1.31	-
	FLM	-	-	1.25	*0.66*	-
	TGM	-	-	*1*	*1*	-
	TMM	-	-	*0.63*	2.13	-
	TMUM	-	-	**0.18**	1.1	-
	FFW	-	-	1.25	1.06	-
	FLW	-	-	**0.50**	1.01	-
	TGW	-	-	1.50	2.1	-
	TMW	-	-	*1*	2.1	-
	TMUW	-	-	*0.65*	1.03	-
	FFE	-	-	-	-	-
	FLE	-	-	*0.75*	*0.66*	-
	TGE	-	-	-	-	-
	TME	-	-	-	-	-
	TMUE	-	-	-	-	-
ESBL *E. coli*	FFM	-	-	-	7.65	-
	FLM	-	-	-	5.33	-
	TGM	-	-	-	1.1	-
	TMM	-	-	-	2.66	-
	TMUM	-	-	-	2.66	-
	FFW	-	-	-	1.33	-
	FLW	-	-	-	*0.66*	-
	TGW	-	-	-	2.66	-
	TMW	-	-	-	2.63	-
	TMUW	-	-	-	2.63	-
	FFE	-	-	-	*0.65*	-
	FLE	-	-	-	2.66	-
	TGE	-	-	-	-	-
	TME	-	-	-	-	-
	TMUE	-	-	-	-	-
*S. aureus*	FFM	-	-	-	-	*-*
	FLM	**0.50**	1.25	1.5	1.33	*1*
	TGM	-	-	-	-	-
	TMM	*0.75*	3	2	22	1.5
	TMUM	*0.75*	3	*0.75*	12	1.5
	FFW	*0.75*	1.50	4	*0.68*	1.5
	FLW	**0.50**	1.5	4	*0.68*	1.5
	TGW	*0.75*	-	1.25	5.26	1.5
	TMW	*1*	5	1.5	42.6	2
	TMUW	*0.75*	3	2	22	1.5
	FFE	-	-	-	-	-
	FLE	-	-	-	-	-
	TGE	-	-	-	-	-
	TME	-	-	-	-	-
	TMUE	-	-	-	-	-
MRSA	FFM	-	-	**0.50**	1.01	-
	FLM	-	-	*0.75*	2.66	-
	TGM	-	-	*1*	21.08	-
	TMM	-	-	*0.65*	10.66	-
	TMUM	-	-	*0.65*	10.53	-
	FFW	-	-	*0.75*	2.66	-
	FLW	-	-	**0.50**	2.63	-
	TGW	-	-	1.5	42.66	-
	TMW	-	-	*0.75*	21.33	-
	TMUW	-	-	*1*	10.66	-
	FFE	-	-	*-*	5.26	-
	FLE	-	-	*-*	1.31	-
	TGE	-	-	*-*	-	-
	TME	-	-	*-*	-	-
	TMUE	-	-	-	-	-
*K. pneumoniae*	FFM	-	-	-	-	*-*
	FLM	-	**0.25**	*0.75*	2.66	**0.18**
	TGM	-	1.5	1.5	21.1	*1*
	TMM	-	*1*	1.25	21.3	*0.75*
	TMUM	-	*0.75*	2	21.3	0.60
	FFW	-	1	*0.75*	0.66	*0.75*
	FLW	-	1	*0.75*	1.03	1.50
	TGW	-	1.50	*1*	42.2	*1*
	TMW	-	2	*0.75*	21.3	*0.75*
	TMUW	-	2	2.5	43	*1*
	FFE	-	-	-	-	-
	FLE	-	**0.37**	*1*	*0.65*	**0.25**
	TGE	-	-	-	-	-
	TME	-	-	-	-	-
	TMUE	-	-	-	-	-
ESBL *K. pneumoniae*	FFM	-	-	-	-	-
	FLM	-	*1*	*1*	2.66	-
	TGM	-	1.50	-	21	*1*
	TMM	-	*1*	-	10.6	-
	TMUM	-	*1*	-	10.6	-
	FFW	-	2	*1*	0.66	-
	FLW	-	**0.50**	*1*	1.03	*1*
	TGW	-	1.50	-	42.16	-
	TMW	-	2	-	42.66	-
	TMUW	-	2	-	10.54	-
	FFE	-	-	-	-	-
	FLE	-	*1*	*0.75*	*0.65*	-
	TGE	-	-	-	-	-
	TME	-	-	-	-	-
	TMUE	-	-	-	-	-

∑ FIC values of plant extracts in combination with conventional antibiotics against sensitive and resistant strains of *E. coli*, *S. aureus*, and *K. pneumoniae*. FF = *Terminalia ferdinandiana* fruit; FL = *Terminalia ferdinandiana* leaves; TG = *Terminalia grandiflora*; TM = *Terminalia microcarpa*; TMU = *Terminalia muelleri*; M = methanol extract; W = water extract; E = ethyl acetate extract; **synergy = ≤0.5**; *additive = >0.5–1.0*; indifferent = >1.0–≤4; antagonistic = >4.0. FIC values were obtained in duplicate. - indicates no inhibition at any concentration tested.

**Table 4:** Qualitative analysis of LC-MS of FLM and FLW.

	Retention Time (Min)	Empirical Formula	Molecular Mass	Putative Identification	Relative Abundance (% Total Area)	
					FLM	FLW
Flavonoids	5.84	C_21_H_20_O_10_	432	Vitexin	2.20	
	6.324	C_15_H_10_O_7_	302	Robinetin	0.06	
	6.322	C_21_H_20_O_12_	464	Quercitin-3β-D-glucoside	0.14	
	6.37	C_27_ H_30_ O_16_	610	Quercitin 3-O-rhamnoside-7-O-glucoside	0.23	
	5.40	C_21_ H_20_ O_11_	448	Orientin	5.42	
	8.53	C_22_H_20_O_12_	476	Hispidulin 7-glucuronide	6.28	
	3.59	C_21_H_20_O_14_	496	Hibiscetin 3-glucoside	0.06	
	5.96	C_21_H_18_O_13_	478	6-Hydroxyluteolin 6-glucuronide	0.06	
	7.07	C_21_H_20_O_11_	448	4-(3,4-Dihydroxyphenyl)-7-hydroxy-5-{[(2S,3R,4S,5S,6R)-3,4,5-trihydroxy-6-(hydroxymethyl)oxan-2-yl]oxy}-2H-chromen-2-one	0.24	
	7.46	C_28_H_36_O_11_	548	3,7-Dihydroxy-4,5-dimethoxy-8-prenylflavan 7-O-beta-D-glucopyranoside	0.70	
	0.32	C_20_H_18_O_13_	466	2-(3,4-Dihydroxyphenyl)-3,5,7-trihydroxy-8-{[(2R,3R,4S,5S,6R)-3,4,5,6-tetrahydroxytetrahydro-2H-pyran-2-yl]oxy}-4H-chromen-4-one	1.47	
	6.19	C_21_H_20_O_10_	432	1,5-Anhydro-1-[5,7-dihydroxy-3-(4-hydroxyphenyl)-4-oxo-4H-chromen-8-yl]hexitol	3.05	
	5.55	C_28_H_24_O_11_	448	(1ξ)-1,5-Anhydro-1-[2-(3,4-dihydroxyphenyl)-5,7-dihydroxy-4-oxo-4H-chromen-8-yl]-D-galactitol	4.74	
	8.77	C_28_H_24_O_12_	552	Vitexin 2″-p-hydroxybenzoate		0.02
	6.30	C_21_H_20_O_11_	448	Orientin		1.65
	4.06	C_16_H_14_O_7_	318	5,7,2′,5′-Tetrahydroxy-6-methoxyflavanone		0.01
	7.38	C_21_H_20_O_10_	432	1,5-Anhydro-1-[5,7-dihydroxy-3-(4-hydroxyphenyl)-4-oxo-4H-chromen-8-yl]hexitol		0.80
	6.97	C_28_H_24_O_15_	600	(2S,3R,4R,5S,6S)-2-{[2-(3,4-Dihydroxyphenyl)-5,7-dihydroxy-4-oxo-4H-chromen-3-yl]oxy}-3,5-dihydroxy-6-methyloxan-4-yl 3,4,5-trihydroxybenzoate		0.40
Tannins	6.09	C_14_H_6_O_8_	303	Ellagic acid (Isomer 1)	3.17	
	7.47	C_14_H_6_O_8_	303	Ellagic acid (Isomer 2)	3.37	2.12
	1.64	C_6_H_6_O_3_	126	Pyrogallol (Isomer 1)		0.47
	0.64	C_6_H_6_O_3_	126	Pyrogallol (Isomer 2)		0.92

## Data Availability

The raw data supporting the conclusions of this article will be made available by the authors on request.
